# The Efficacy of Selected Desensitizing OTC Products: A Systematic Review

**DOI:** 10.1155/2014/865761

**Published:** 2014-03-27

**Authors:** E. Talioti, R. Hill, D. G. Gillam

**Affiliations:** ^1^Centre for Adult Oral Health, Bart's and the London Queen Mary's School of Medicine and Dentistry, Queen Mary University of London (QMUL), Turner Street, London E1 4NS, UK; ^2^Dental Physical Sciences Unit, Barts and the London School of Medicine and Dentistry, QMUL, London, UK

## Abstract

*Objectives*. The aim of the present study was to review the published literature in order to identify relevant studies for inclusion and to determine whether there was any evidence on the clinical effectiveness of selected desensitizing toothpastes, calcium sodium phosphosilicate (CSPS), amorphous calcium phosphate (ACP), nanohydroxyapatite, and casein phosphopeptide-amorphous calcium phosphate (tooth mousse) on reducing dentine hypersensitivity (DH). *Materials and Methods*. Following a review of 593 papers identified from searching both electronic databases (PUBMED) and hand searching of relevant written journals, only 5 papers were accepted for inclusion. *Results*. Analysis of the included studies (3 CSPS and 2 ACP) would suggest that there may be some benefit for patients using these products for reducing DH. No direct comparative studies were available to assess all these products under the same conditions neither were there any comparative randomised controlled studies that compared at least two of these products in determining their effectiveness in treating DH. * Conclusions*. Due to the small number of included studies, there are limited clinical data to support any claims of clinical efficacy of these OTC products. Further studies are therefore required to determine the efficacy of these products in well-controlled RCT studies with a larger sample size.

## 1. Introduction 

Dentine hypersensitivity (DH) is a worldwide clinical condition that has been reported to have an impact on the adult population at various stages during their lifetime. Recently, several investigators [1, 2] reported on the negative effects of DH on a patient's quality of life (oral health-related quality of life, OHRQoL). The prevalence of DH reported in the literature varies depending on the methodology utilized to collect data; however, it has been reported to affect up to 74% of the population although several investigators have reported that this figure may be higher in individuals with periodontal disease [3, 4]. According to a number of investigators [5, 6], DH appears to have been previously underreported by patients as well as underdiagnosed by dentists which therefore may lead to the problem being ignored and undertreated. Diagnosis of DH can be problematic but from the clinician's viewpoint, it is essential to exclude all other clinical conditions that have a similar pain history.

The management of generalized mild or moderate DH has traditionally been achieved via the use of over-the-counter (OTC) desensitising products through a clinician's recommendations which depend on the nature of its active ingredient that may take up to 2–4 weeks to achieve some resolution of the pain associated with DH. There is a plethora of remedies currently available for both OTC and in-office alleviation of DH, however none of these products appear to be the “gold standard” in providing a long term effective treatment to the problem [5, 7, 8]. Marketed products currently work either via their tubule occlusion properties, for example, calcium sodium phosphosilicate (CSPS [Novamin]), amorphous calcium phosphate (ACP), nanohydroxyapatite (HAP), and tooth mousse or via nerve desensitization, for example, potassium technology [7, 8].

One of the problems however in evaluating the reported efficacy of both OTC and in-office products was that while there were reported claims of either immediate or long lasting effectiveness, there were limited clinical data to support these claims under standardized clinical procedures.

## 2. Aims and Objectives

The aim of the present study was to critically review the available published literature in order to identify all relevant studies for inclusion and to determine whether there was any evidence on the clinical effectiveness of selected over-the-counter (OTC) toothpastes, calcium sodium phosphosilicate (CSPS [Novamin]), amorphous calcium phosphate (ACP), nanohydroxyapatite (HAP), and tooth mousse dentifrices on reducing dentine hypersensitivity (DH).

## 3. Methodology

The search methodology used for the current review was a modified version of the Cochrane systematic review by Poulsen et al. [9] and Karim and Gillam [7].

### 3.1. Selection Criteria

#### 3.1.1. Types of Study

This review will include all full text, double-blinded, randomized controlled trials (RCT) conducted* in vivo* to assess the efficacy of any of the desensitizing agents named above and their effect on DH. The duration of the included studies should be at least 6 weeks in duration.

#### 3.1.2. Types of Subjects

The subjects included in the relevant studies are dentate, healthy adults (of at least 18 years of age) with a reported and established DH diagnosis. Studies were excluded if the sample in the original study was not described or if the subjects included into the study had received periodontal treatment within the period of the trial or if the participants are/were undertaking anti-inflammatory treatment due to medical problems. The number of participant dropouts and reason for dropout should be included in the study.

#### 3.1.3. Types of Outcome Measurement

Assessing DH at baseline and after treatment with a desensitizing agent would be an ideal way of comparing data and DH prevalence reduction if any. DH can be measured via numerous methods, for example, tactile, thermal or evaporative stimuli, patient questionnaires, and so forth. The included studies will incorporate a detailed explanation of

- history of DH as assessed at baseline data by at least one recognized methodology (tactile, thermal);
- history of DH as assessed following desensitizer use.

#### 3.1.4. Types of Intervention

The participants were randomly allocated to individual groups receiving one of the following:

- test desensitizer (% concentration of desensitizer should be stated by the authors);
- negative control (same as test group however lacking the active ingredient (minus active ingredient)).

The ideal negative control group would entail the usage of a toothpaste of the same composition as the test desensitizer however lacking the active ingredient under test. Studies were included if fluoride was absent or present (at same concentration) in both groups. Studies were excluded if the test toothpaste contained fluoride whereas the control did not. Studies were accepted into the present review if the above criteria of both groups (a) and (b) were met; other groups (e.g., groups C, D, etc.) testing different percentage of desensitizers were acceptable provided that a negative control was established.

#### 3.1.5. Other Relevant Criteria

- Investigator calibration on assessment of DH.
- Statistical analysis.
- Randomization of the participants into different groups was clearly described, concealment of participant group allocation to both investigators and subjects.

## 4. Search Strategy

The search strategy was included using electronic databases (e.g., PUBMED) and hand searching up to 31, December, 2012. Hand searching included examining the relevant published or incomplete journals in English. The searching keywords in PUBMED were (dentifrice OR dentifrices) OR (toothpaste OR toothpastes OR tooth paste) OR (desensit*) AND (agent OR efficacy OR effect) AND (dentin OR dentine or tooth OR teeth OR root*) AND (Hypersensitivity OR hypersensit* OR sensitivity OR sensitiv∗ OR over-sensit*) NOT (laser* OR adhesiv* OR endodont* OR bleach* or whitening OR bond* OR caries)

- AND (hydroxy apatite OR nanohydroxyapatite OR nano HAP OR nHAP).
- AND (casein phosphopeptide amorphous calcium phosphate OR CPP-ACP).
- AND (amorphous calcium phosphate OR ACP).

## 5. Statistical Analysis

Statistical analysis of the data from these studies was not attempted due to the variations in the study design, methodology, study duration, and reporting of the pain response (percentages, VAS scores, or pain categories, etc.).

## 6. Method of the Review (Data Collection and Analysis)

A review of the abstracts and titles was carried out by one of the reviewers (E. T.) who then obtained copies of all the relevant studies where available. Two reviewers (E. T. and D. G.) subsequently sought to determine the eligibility of the papers and data extraction. Any differences as to the inclusion or exclusion of articles were resolved following discussion between the two reviewers.

### 6.1. Quality Assessment of the Included Studies

The methodological quality of the studies included in the review was assessed according to the criteria of concealment of treatment allocation described in the Cochrane Handbook for Systematic Review of Intervention [10].

The acceptance and rejection criteria for the inclusion of relevant studies for the present review were discussed between the two reviewers (E. T. and D. G.) prior to the collation of papers.

## 7. Results

### 7.1. Overall Description of the Included and Excluded Studies

**Figure 1:**
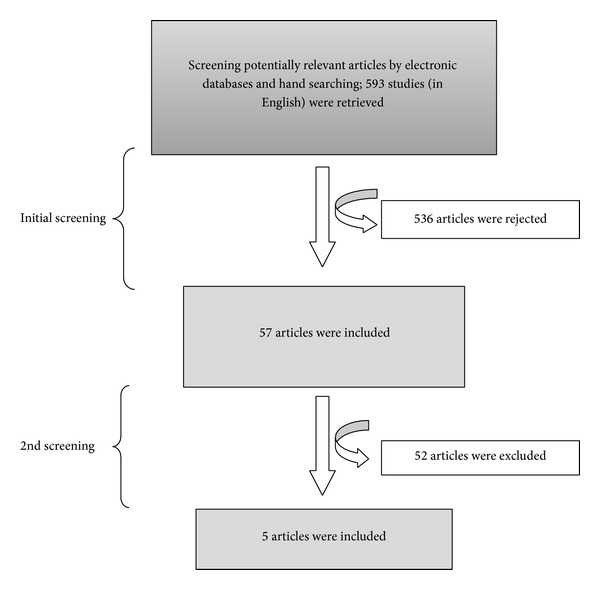
The flow diagram of the study selection process.

Following the initial screening of identified articles for the present review, there were 593 potentially relevant studies found by searching either the electronic databases (PUBMED-574 titles) or by hand searching (19 relevant titles) articles from the literature. Unpublished studies were found by searching the electronic databases or by hand searching. 57 studies were regarded as relevant for this review while 536 were excluded. The 57 studies were grouped into Novamin papers comprising of 34 studies, ACP papers comprising 10 studies, HAP papers comprising of 11 studies, and CPP-ACP/tooth mousse papers comprising of 2 studies (Figure 1).

Following an evaluation of the various papers, of the 34* Novamin* studies, 31 studies were excluded and 3 were included (Table 1), of the 10* ACP* relevant studies, two studies were included and eight studies were excluded (Table 2), of the 11* HAP* relevant studies, all studies were excluded (Table 3), of the 2* CPP-ACP* relevant studies, all studies were excluded (Table 4).

### 7.2. Excluded Studies

#### 7.2.1. Novamin Studies

There were 15 studies which were excluded as these were* in vitro* investigations and therefore did not meet the inclusion criteria [11–25]. Of the 19 remaining studies, one review was also excluded [26] and three were excluded as they were reported as an abstract [27–29]. One study was excluded as the full published article was in Chinese and only the abstract was in English [30]. Two further studies were excluded as Novamin was used as an in office agent (desensitizing polishing paste) and not as an OTC desensitizing toothpaste [31, 32]. One study was excluded due to its duration of ≤10 days [33] and a further study was excluded due its aims; for example, Tai et al. [33] investigated the antigingivitis effect of Novamin on the gingival tissues (and not as a desensitizing toothpaste* per se*) and therefore the study was considered irrelevant to this review. Of the remaining ten studies, three were excluded as the publication date was after December 31st [34–36]; the Neuhaus study [37] evaluated a In office professionally applied polishing paste, whereas the other two studies lacked a negative control in the clinical trial design methodology [35, 36]. Two further studies were excluded since the negative control group contained fluoride whereas the test group (Novamin) did not [38, 39]. One study was excluded because the dentine specimens were placed in intraoral appliances worn by patients to establish an* in situ* effect of Novamin [40]. One further study was excluded due to the lack of description on the composition of the control randomization [41] (Table 1).

#### 7.2.2. ACP Studies

Four studies were excluded as these were* in vitro* investigations examining the effects of ACP on dentine tubule occlusion [42–45]. One study was excluded since the control group contained sodium fluoride (NaF) and the test group did not [46]. The Giniger et al. [47] study was excluded due to the investigators examining DH as a result of vital bleaching which may be considered to be a distinct mechanism from DH [48]. Geiger et al.'s [49] study was excluded due to the lack of a negative control (the control group was a Potassium Chloride (KCl) product) and the final excluded study by Yates et al. [50] was excluded since the ACP was applied as an in-office agent and not as an OTC dentifrice (Table 2).

#### 7.2.3. HAP Studies

There were eight studies which were excluded because they were* in vitro* investigations and did not meet the inclusion criteria [51–58]. Another study was excluded since the HAP was used as an in-office agent rather than a toothpaste [59] whereas Orsini et al.'s [60] study was excluded due to the lack of a negative control being used. The final excluded study [61] was excluded due to the investigators examining the effect of HAP on DH following dental bleaching, which as previously indicated was suggested to have a different mechanism to that described for DH [48] (Table 3).

#### 7.2.4. CPP-ACP Studies

Two studies were excluded since these were* in vitro* investigations investigating the effect of CPP-ACP on dentine tubule occlusion [62, 63]. No other studies related to DH were retrieved as a result of the search strategy for the present review (Table 4).

### 7.3. Analysis of the Included Studies 

#### 7.3.1. Study Design

The five included studies in the present review comprised of randomized controlled trials (three for Novamin and two for ACP) (Tables 5 and 6). Pradeep et al. [64] was a triple-blinded, randomized controlled trial. Randomization was generated via a random computer table and the investigators were not involved in this procedure to ensure their blinding. Participants, investigators, and statisticians were blinded and the toothpastes were placed in white tubes labelled A, B, C, and D so that allocation concealment was established. The Pradeep and Sharma [65] study was also a triple-blinded randomized controlled trial. Triple blindness was established via dispensing toothpastes in identical tubes which were coded. The true identity of each tube was only confirmed (breaking of the codes) following the final clinical assessment by these investigators. The Litkowski and Greenspan [66] study was a double-blinded randomized controlled trial; however, details on randomization of both subjects and products were not provided in the paper.

Ghassemi et al. [67] stratified their subjects into VAS, age, and gender and then randomly allocated them into three groups which were representative of the sample. Allocation concealment was achieved by dispensing toothpastes in identical tubes, the identity of which was not revealed to participants or investigators until the end of the study. No details were provided as to how and by whom the randomization and allocation concealment process was conducted.

The Kaufman et al. [68] study was a double-blind randomized controlled trial; subjects were stratified according to prognostic measures, tactile scores for sensitive teeth, the number of sensitive teeth per subject, and the proportion of sensitive teeth per subject. Subjects in each group were then provided with one of the three treatment modalities.

#### 7.3.2. Study Population

The participants fulfilling the inclusion criteria for Pradeep et al. [64] and Pradeep and Sharma [65] reported a history of DH as a result of subjects exhibiting either gingival recession or cervical abrasion. Subjects were required to have at least two teeth with a VAS of 4 or more in order to be included into the study. Teeth included were included provided that they had either small or no occlusal restorations. Teeth with caries, defective restorations, and orthodontic appliances or bridgework were excluded. Participants for these two studies were also excluded if they were allergic to any of the ingredients of the toothpastes tested or suffering from chronic diseases, oral pathology, and eating disorders for which they were taking anti-inflammatory medications or analgesics.

The former paper recruited 160 subjects; however, only 149 completed the study; 72 males and 77 females. The latter paper recruited 120 participants of which 110 completed the study; 58 males and 52 females.

Litkowski and Greenspan [66] recruited healthy male and female adults suffering from DH who were not using a desensitizing agent at the time of the study. Participants suffering from medical conditions, taking medications which may interfere with pain perception or response, or allergic to any of the toothpastes used in the study were excluded. A total of 66 subjects were recruited in the study however no details were forthcoming regarding gender or age of subjects.

Ghassemi and coworkers [67] recruited 208 participants in order that at least 100 subjects in each group would complete the study. The inclusion and exclusion criteria were clearly described in the study; subjects with severe periodontal disease, gross oral neglect, periodontal, restorative, or orthodontic treatment within the last three months or dental prophylaxis within the last two weeks. Subjects were also excluded if they were suffering from postbleaching tooth sensitivity or were taking medications which would interfere with pain perception.

Kaufman et al. [68] recruited 105 healthy subjects with at least one sensitive tooth to either scratching with a dental explorer, a one-second air blast, or both stimuli. Teeth with caries, cracks or large restorations were excluded; subjects who have used a desensitizing toothpaste within the last 6 weeks prior to the study were also excluded. Kaufman and coworkers [68] however only examined canines and premolars for inclusion in the study.

#### 7.3.3. Age Range of Participants

Participants' age ranged from 20 to 60 years of age with a mean of 39.9 years [64]. In the study by Pradeep and Sharma [65] the age ranged between 20 and 60 years with a mean of 39.4 years. Litkowski and Greenspan [66] however did not state the age range of the subjects recruited in their study. Ghassemi et al. [67] included patients within 18–64 years of age. The Kaufman et al. [68] study did not mention the age range of participants.

#### 7.3.4. Study Duration

The ideal duration for most clinical trials assessing the efficacy of a desensitizing agent has been considered to be eight weeks; however, Holland et al. [69] reported that the “*optimum time course for different agents, differs based on their action.*” Studies were of similar duration ranging from six to eight weeks; Pradeep et al. [64] and Pradeep and Sharma [65] studies lasted six weeks and the DH assessment was recorded at baseline, two, and six weeks. The Litkowski and Greenspan [66] study lasted eight weeks and DH assessment was conducted at baseline, two, four, and eight weeks posttreatment time intervals. Ghassemi et al. [67] study was a two-phase trial in which each phase lasted eight weeks; DH assessments were conducted at baseline, four, and eight weeks. Kaufman et al. [68] was an eight-week long trial; DH assessments were recorded at baseline, three, and eight weeks after treatment (Table 7).

#### 7.3.5. Statistical Power

Sample size calculations in the Pradeep et al. [64] and Pradeep and Sharma [65] studies were performed according to a 30% VAS reduction between test and control groups with a two-tailed significance level of 5% and a power of 90%. Litkowski and Greenspan [66] estimated a sample size of no more than 10 was required to provide with a 40% relative efficacy for a test product over a placebo; this was calculated via a two-tailed alpha of 5% with 80% power. Ghassemi et al. [67] calculated that a sample size of at least 100 subjects in each group would provide a power of 80% for detecting a significant difference of 30–35% between group mean VAS scores. There was no mention of the statistical power in the Kaufman et al. [68] study.

#### 7.3.6. Randomization and Allocation Concealment

According to Schulz [70], random allocation to intervention groups in a clinical study appears to be the only method of ensuring that the groups being compared have an equivalent foothold at study outset hence eliminating confounding factors or the introduction of bias into the study. Of the three included Novamin randomized studies (RCT) studies, only two studies [64, 65] reported on details on randomization and allocation concealment. For example, Pradeep et al. [64] used a computer-generated random table to allocate participants into four different groups. The investigators of this study were not involved in the randomization process as the study statisticians were responsible for the allocation of participants into groups and as a consequence were blinded. This study was therefore considered to be triple-blind in nature and hence the treatment received by each group was concealed to everyone involved in the study, namely, investigators, participants, and statisticians. The codes were only broken following the completion of the study. Pradeep and Sharma [65] randomly allocated participants into the three treatment groups via a lottery method; however, no further explanation or details were forthcoming from the study. Participants and investigators however were blinded as to the contents of the three identical tubes A, B, and C; contents of each tube were subsequently revealed to the investigators following the last assessment. Litkowski and Greenspan [66] failed to disclose any information as to the randomized or allocation concealment between the different groups in the study. Ghassemi et al. [67] stratified individuals according to VAS scores, gender, and age; randomization into the three groups was carried out according to the strata created so that three equivalent groups were formed. No detail was provided regarding who carried out the randomization and allocation concealment. Kaufman et al. [68] did not mention any methods of randomization or allocation concealment.

#### 7.3.7. Consideration of Withdrawals and Dropouts

According to Bowers [71] withdrawals and dropouts that may occur following the randomization process may affect the balance of the groups established via the randomization procedure. One way of avoiding this problem is by reporting on the number of withdrawals or dropouts as if they were still a part of the clinical trial; this is called the* intention-to-treat analysis*. Withdrawals and dropouts were reported in all three included studies; Pradeep and Sharma [65] reported 10 out of 120 whereas Pradeep et al. [64] reported 11 out of 160 dropped out of the trial; both studies reported the reason of withdrawal was that participants failed to follow up or discontinued treatment. Litkowski and Greenspan [66] reported no dropouts. Ghassemi et al. [67] did not report on any dropouts during the first phase of the trial; however, carefully going through the results, it can be observed that five participants dropped out. During the second phase of this trial, thirteen participants decided to discontinue the treatment; no reason was provided by authors. Kaufman et al. [68] reported four dropouts during the duration of the trial; the authors however did not report the reason for withdrawal, but it was recorded that there were no adverse side effects reported by any of the participants (Table 8).

## 8. Data Analysis

No further analyses were performed on the mean differences from 6 to 8 weeks for any other measurement outcomes for the purpose of meta-analysis.

### 8.1. Previous History of DH Reported at Baseline

This included any history of DH in the included studies, reported by investigators, in the form of baseline data, which was confirmed by a response to tactile and/or thermal stimulus. Minor data analysis variations existed between Pradeep et al. [64] and Pradeep and Sharma [65]; both studies reported on the mean VAS of each group at baseline, two, and six weeks in the presence of air and water stimuli. Pradeep et al. [64] used a one-way analysis of variance (ANOVA) and *P* < 0.05 was considered to be a statistically significant difference when detected. Pradeep and Sharma [65] also used post hoc one-way analysis of variance; however, the Holm-Sidak method was used (*P* < 0.05). Litkowski and Greenspan [66] also reported on the % reduction of DH from baseline and included the mean +/− standard error change from baseline. These investigators also used a one-way analysis of variance (ANOVA test) on the change of VAS from baseline at two, four, and eight weeks. Duncan's multiple range test was used to rank group differences if the treatment factors were found to be significant at alpha being equal to 0.05. Ghassemi et al. [67] presented their results in tabulated and graphical form which allowed the reader to make the necessary conclusions from the study. The primary outcome was determined by comparison of mean VAS scores at the three-interval visits; analysis of variance (ANOVA) was used to compare baseline mean VAS scores. Within group differences were calculated using student's *t*-test for paired data at four and eight weeks. Differences between the different groups were calculated using the analysis of covariance (ANCOVA) model. The secondary outcome of the latter study was determined via the mean Schiff Thermal Sensitivity Scale score (STSS) at each of the three exam intervals. Mean STSS scores were used to calculate Wilcoxon's signed rank test; this statistical test was used to evaluate within group differences. Wilcoxon's rank sum test and chi-squared test were used to calculate differences amongst the different groups. Kaufman et al. [68] presented their results in tabulated form comparing the total number of teeth positive and negative for DH at baseline, three, and eight weeks when assessed using tactile or air stimuli. A graphical representation of results was also depicted via combined line graphs to indicate the difference between the three groups. A logit transform of the proportion of the examined teeth (testing positive for DH) was calculated; within group differences were calculated via a 2-way analysis of variance and *t*-tests with the Bonferroni correction were evaluated between group differences. The Kruskal-Wallis, Friedman's 2-way analysis of variance, and chi-squared tests were used to analyse the questionnaire results for the various time intervals.

### 8.2. Types of Treatment Intervention

In all the 5 included studies, a daily home use of calcium sodium phosphosilicate (Novamin) (Pradeep et al. [64], Pradeep and Sharma [65], Litkowski and Greenspan [66]) and/or ACP (Ghassemi et al. [67], Kaufman et al. [68]) (Tables 5 and 6). No studies for HAP and CPP-ACP toothpastes however were included in the present review (Tables 3 and 4).

### 8.3. Clinical Methodology Used to Assess DH

The most commonly used method of clinically assessing DH in these three studies was an evaporative stimulus. This was conducted using a controlled air pressure from a standard dental syringe at 40–65 psi perpendicular to the tooth surface at a distance of 1–3 mm [64, 65]. Litkowski and Greenspan [66] using similar methodology reported a blast of cold air for one second. The second most popular method of DH assessment was a thermal stimulus via the application of 10 mL of ice cold water to the exposed root surface [64, 65]. Litkowski and Greenspan [66] reported on using a tactile stimulus perpendicularly to the long axis of the tooth and directly onto the exposed root surface. A Yeaple probe (XINIX Inc., Portsmouth, UK) was calibrated to deliver 40 g force along the root surface when assessing DH. Ghassemi et al. [67] used a thermal stimulus of a cold air blast on exposed root surfaces and subsequently recorded a VAS and Schiff score.

Kaufman et al. [68] assessed DH at baseline, three, and eight weeks following the use of the three different treatment modalities. Subjects were provided with a questionnaire at each visit and were asked to rate their sensitivity as none (0), mild (1), moderate (2), or severe (3). These investigators used electrical, tactile, and air stimuli; the latter was used last due to its ability to cause a prolonged painful response. To evaluate an electrical stimulus, the sensitometer was used; its tip was moistened with a conducting gel and then placed in the midline of the enamel surface at the gingival 1/3 of the tooth under test. The voltage was slowly increased until the subject experienced a sensation at which point the stimulus was stopped via a button controlled by the participant. Tactile stimuli were assessed via the scratchometer (SUNY Stony Brook, Stony Brook, NY). The stainless steel explorer tip was moved mesiodistally along the CEJ at a fixed pressure until the participant experienced sensitivity or if the pressure had exceeded 80 centi-Newtons. The last stimulus used was a response to a one-second blast of air from a dental air syringe held 0.5 cm from the centre of the tooth. The participants were asked to rate their response to the stimulus as 0—no discomfort, 1—uncomfortable, 2—painful, or 3—painful with persisting pain after stimulus was ceased. The three different stimuli measured DH at the three and eight week assessments; at baseline following the screening of suitable patients and teeth, a DH assessment (by air and tactile stimuli) was conducted on the selected teeth and patients were subsequently accepted into the study.

### 8.4. Calibration and Examiner Training

Ghassemi et al. [67] reported that their investigators were calibrated regarding the DH assessment tests within the last year. None of the other four studies stated or reported any calibration taking place amongst clinicians [64–66, 68].

### 8.5. Compliance

Only two of the five papers reported on compliance, for example, Ghassemi et al. [67] monitored compliance by weighing the toothpaste at baseline, 4, and 8 weeks and by monitoring the subjects' diaries (daily tooth brushing regime including timings). The Litkowski and Greenspan [66] study reported compliance by weighing the toothpaste over the 8-week period; for example, the control group used 142.4 g of toothpaste over the 8-week period whereas participants in the 2.5% and 7.5% groups used 161.5 g and 144.5 g of toothpaste, respectively.

## 9. Discussion 

Dentine hypersensitivity (DH) is a common dental complaint which may have a profound effect on an individual's quality of life [1, 2, 72]; however, DH tends to be an underestimated condition due to underreporting by its sufferers and also the difficulty in diagnosing it [6]. Pain experienced and reported due to DH is subjective in nature and depends primarily on the individuals' previous experiences of pain; this can create discrepancies in clinical trials involving DH prevalence. Evaluating the latter for the purpose of clinical trials can be complicated due to both the Hawthorne and placebo and nonplacebo effects throughout the duration of the study [73]. The true placebo response is seen in participants whose pain response or perception may change over a period of time especially if they were participating in a clinical trial testing for a desensitizing agent; these subjects are normally unaware that they have been randomized in the control group. The placebo effect has been reported to range from 20–60% from the baseline measurements [74, 75]. The Hawthorne effect can be described as the unconscious change in participant behaviour due the mere knowledge of being observed during a clinical trial. Bias can be introduced into clinical trials as a result of a number of reasons: lack of statistical power, for example, small sample size or lack of standardization of the methodology, no allocation concealment, or improper stratification and randomization of groups.

There is a plethora of remedies currently available for both OTC and in-office alleviation of DH; however, none of these products appear to be the “gold standard” in providing a long term effective treatment to the problem [3, 5, 6]. Marketed products currently work either via their tubule occlusion properties, for example, Novamin, ACP, HAP, CPP-ACP, toothpastes and so forth, or via nerve desensitization, for example, potassium technology.

The current review examined the available published literature (in English) on four marketed agents currently being used to alleviate dental problems, namely, calcium sodium phosphosilicate, (nano)hydroxyapatite crystals, amorphous calcium phosphate, and casein phosphopeptide amorphous calcium phosphate (now GC Toothmousse) toothpastes. Five clinical trials were included following an extensive review of the published literature description up to 31, December, 2012, three on calcium sodium phosphosilicate [64–66] and two studies on ACP [67, 68]. None of the available literatures on (nano)HAP or CPP-ACP were included in this review as previously indicated (Tables 3 and 4).

One of the problems when evaluating the efficacy of the selected products for the present review was that in retrospect the criteria may have been too restrictive when considering the studies with matched placebo controls. It may therefore be argued that the inclusion of studies with a valid negative control, for example, the same constituents as the agent under test without the active ingredient, would have allowed the authors to make an evaluation among two or three comparable groups and hence any changes or improvements in DH would be attributed to the active ingredient under test. However, the rationale for conducting this review was to determine whether the active ingredient in the tested toothpastes delivers efficacy in the reduction of DH which is the basis of the claims made for these toothpastes by the manufacturers [7]. Other benefits attributed to these toothpastes such as antiplaque, anticaries, and reduction in DH following vital tooth bleaching have not been considered in this review.

Of the 34 relevant Novamin studies that were retrieved by the authors, the majority of these studies were excluded [11–41] (Table 1). only three RCTs were included into the present review; Pradeep et al. [64], Pradeep and Sharma [65], and Litkowski and Greenspan [66].

Pradeep et al. [64] conducted a triple-masked randomised controlled trial to evaluate and compare the efficacy of (a) 5% potassium nitrate (KNO_3_) (positive control), (b) 5% calcium sodium phosphosilicate (test), (c) 3.85% amine fluoride, and (d) placebo (negative control) toothpastes on DH alleviation. The randomisation of groups was conducted via computer generation and investigators, participants, and statisticians were all blinded as to which treatment each group they would be allocated to. The toothpastes were dispensed in identical white tubes which were then labelled as A, B, C, or D; the identity of each tube was revealed to the investigators only after the last assessment had taken place. This process was conducted in this manner to maintain allocation concealment and to prevent the introduction of any bias or confounding factors in the trial. Sensitivity assessment was carried out via evaporative (air blast) and thermal (10 mL of ice cold water) stimuli. Participants were instructed on how to use the VAS (0–10 cm) and their self-reported DH assessment data were analysed by the statisticians. Assessments were carried out at baseline, two, and six weeks after treatment and statistically significant improvements in DH were observed in all four groups; the most significant improvement was observed in the calcium sodium phosphosilicate group. KNO_3_ was used as a positive control due to its universal market availability (particularly in the USA). Fluorides such as NaF or stannous fluoride have been previously shown to be effective in the treatment of DH via their ability to deposit calcium fluoride and occlude the patent dentine tubules. The placebo group of individuals may also respond positively to the treatment; the reasons for this may be either placebo or Hawthorne effects or even the participants' desire to impress or please the investigators and regression to the mean or mode.

Pradeep et al. [64] failed to report on any calibration among participants and their recording of DH which would be an important part of this trial due to the subjectivity of pain. The authors did not disclose any information as to how many investigators or statisticians took part in the study and statistical analysis followed was somewhat unclear. One of the limitations of this study was the lack of description of the constituents of the placebo group. It would therefore be logical to assume that the placebo control may be one of the following: no treatment, distilled water, or even the same constituents as the test groups but lacking the active ingredients. If the latter was the true constituent of the placebo group, then this RCT was correctly included into this review.

Pradeep and Sharma [65] conducted a triple-masked randomization controlled clinical trial to evaluate and compare the efficacy of (a) 5% calcium sodium phosphosilicate, (b) 5% potassium nitrate (positive control), and (c) toothpaste with same contents as (a) but without the active ingredient (negative control) on DH alleviation. Participants were allocated into individual groups by a lottery method; however, no information was obtained from the full text of this study as to who carried out the randomization and how blinding of investigators had taken place. The toothpastes were dispensed in tubes labelled as A, B, and C; the contents of which were disclosed to the investigators after completion of statistical analysis. Sensitivity assessments were conducted using the same methods as in Pradeep et al. [64] at baseline, two, and six weeks after treatment and hence the same limitations were introduced due to the limited duration of the study (6 weeks). Statistically significant improvements to DH were observed in all three groups; the CSPS group exhibited the most significant improvement out of all three treatment modalities. The changes observed in the placebo group were again attributed to the placebo or Hawthorne effects. As previously mentioned, the fact that the inclusion of a negative control of the same composition as the test toothpaste but without the active ingredient was used enabled the authors to make valid conclusions (given that the groups involved appeared to be comparable in all aspects) that any changes in DH could be attributed to the active ingredient in group (a). No information however was available on any calibration between the investigators and the statistical analyses used did not appear to be consistent with good statistical practice.

Litkowski and Greenspan [66] in their first pilot study for CSPS randomly allocated 66 participants into three groups. The investigators claimed that this trial was double-blinded in nature; however, no information was available to determine how the investigators and volunteers were blinded. There were no details on allocation concealment and this could potentially introduce bias and confounding factors. The aim of this study was to evaluate and compare the efficacy of (a) 2.5% CSPS, (b) 7.5% CSPS, and (c) a placebo control on DH management. DH assessments were conducted using tactile (Yeaple probe) and evaporative (one second blast of air) stimuli at baseline, two, four, and eight weeks after treatment. The results were presented in a tabulated form; the 7.5% CSPS group exhibited the greatest improvement in DH from baseline whereas placebo and 2.5% CSPS exhibited similar % improvement at the end of the 8-week period. Limitations involved with the Litkowski and Greenspan [66] study involved (a) the method of randomization; how did this take place and who carried it out, (b) blinding of investigators and participants was not described, (c) no description was given regarding the volunteers' age range, M : F ratio, (d) no calibration methods were mentioned, (e) statistical analyses did not conform with good statistical practice, and (f) reason for choosing particular sample size was not mentioned and the eligibility criteria were not clearly described to the reader. The most important discrepancy in this RCT would have to be the lack of description of the constituents of the placebo group; the authors omitted this information in their study and hence groups a and b could not potentially be comparable to group c. The reason for inclusion of this RCT into the present review was initially in doubt due to the lack of clarity of the control group; it may be speculated that the placebo control group consisted of the same constituents as the test groups but without the active ingredients.

Of the included studies, only the Litkowski and Greenspan [66] study recorded any patient compliance during the clinical trial and this was by weighing the toothpaste before and after usage by the patients.

One of the problems in evaluating the efficacy of the Novamin dentifrice or toothpaste was that there are limited published clinical data that consistently supported the product. For example, in the included studies, Litkowski and Greenspan [66] examined two different concentrations, namely 2.5% and 7.5% bioglass against a placebo (0% bioglass). A 5% Novamin version is currently on the market even though that Litkowski and Greenspan [66] study reported that the 7.5% Novamin was superior to 2.5%. There does not appear to be any published clinical studies comparing 5% to 7.5% Novamin. Furthermore, the formulations in the three studies and the current formulation are different; for example, there was no fluoride in the earlier formulations of Novamin although a later formulation included 0.24% stannous fluoride and the current commercially available Novamin contains a sodium monofluorophosphate (MFP) (1450 ppm). The different fluoride ingredient and concentration of Novamin in the currently commercially available version may be due to either regulatory or formulation issues. One of the problems in formulating a toothpaste containing Novamin is related to its ability to absorb moisture from the atmosphere and the current formulation is anhydrous in nature.

Most of the ACP studies retrieved (apart from the 2 included studies) were excluded as previously indicated in Table 2. Of the 10 studies, four were* in vitro* investigations examining the effects of ACP on dentine tubule occlusion and hydraulic conductance [42–45]. randomized trials, for example, the Fiocchi et al. [46] and Geiger et al. [49] studies, lacked negative controls and Giniger et al. [47] investigated the remineralization potential of ACP following vital tooth bleaching and the study was subsequently excluded. Yates et al. [50] conducted a double-blind randomized split mouth study and applied intraoral ACP to one side of the oral cavity and a control dentifrice to the contralateral side. All applications were conducted in office and the results demonstrated that the test (ACP) toothpaste did not provide any significantly superior results compared to a control toothpaste. Of those studies included in the present review, Ghassemi et al.'s [67] study was a double-blind, two-phase RCT which fulfilled all the criteria of the present review; the duration was longer than 6 weeks and the sample size was sufficient to provide a significant result; the examiners were also calibrated prior to the study, and the test group contained the ingredient under investigation (delivered calcium, phosphate, and 0.24% NaF) whereas the control group delivered an identical level of NaF to the participants. During the first 8-week phase of the trial, 208 participants were stratified according to their VAS scores, gender, and age; they were then randomly assigned to either the ACP (plus 0.24% NaF) group or control group (0.24% NaF). Allocation concealment was achieved via dispensing both products into identical white coded tubes; however, there was no detail as to who carried out the concealment and randomisation of groups. VAS and Schiff scores were recorded at baseline, four, and eight weeks. During the second phase of the trial, subjects from the test group were recruited for a second 8-week study to assess the persistence of DH via the home use of the control toothpaste (90 of 103 subjects took part). The authors concluded that the test toothpaste provided superior results when compared to the control (fluoride only) group; however, DH improvement had also been observed in the control group and this again was attributed to the placebo or Hawthorne effects.

Kaufman and coworkers [68] conducted a double-blind randomized clinical trial; however, the authors did not report on how the randomization and allocation concealment was conducted. There was no mention of the participant age range and there were significantly higher proportions of females compared to males (72% versus 28%). The authors screened participants for DH by tactile and evaporative stimuli; however, they recorded DH at three and eight weeks using electrical, tactile, and evaporative stimuli. One of the problems with this study particularly when compared to the other included studies was that the electrical and mechanical stimuli used in the assessment were used solely by the Kauffman/Kleinberg group at Stony Brook University, USA, and rarely used outside that Institute, and the other studies recorded using similar tactile and thermal stimuli. The use of an electrical stimulus in this type of student has been questioned as it is based on voltage and not constant current which has been considered more appropriate if using an electrical stimulus [73]. Furthermore, the results were presented in tabulated form for tactile or air stimuli; the authors did not provide sufficient information on either the air or tactile stimuli and therefore any comparison with intraoperative results would be problematic. The three treatment groups were also unclear; the authors did not mention the constituents of group A which acted as the control group or the % of fluoride present. These observations may therefore limit the conclusions related to the efficacy of the products tested in this study.

There were eight nano-HAP studies which were excluded because they were* in vitro* investigations (effect on dentine tubule occlusion) and therefore did not meet the inclusion criteria [51–58] (Table 3). Two randomized controlled trials [51, 52] investigating the effects of nano-HAP on DH were also retrieved and subsequently excluded from the present review due to the fact that (1) the desensitizing agents were applied in office and as a dry sol gel or liquid precipitate form and not as a OTC dentifrice or toothpaste [59] and due to (2) the lack of a negative control respectively [60]. A further study by Browning et al. [61] investigating the effect of HAP in DH attributed to dental bleaching was also excluded from the present review as this mechanism has been considered to be a completely distinct mechanism to DH [48].

CPP-ACP also known as GC tooth mousse has been used for remineralizing a demineralized lesion on the tooth surface. The two studies retrieved for the purpose of the present review (Gandolfi et al. [62] and Ranjitkar et al. [63]) were excluded as they were* in vitro* studies (Table 4).

## 10. Conclusions

One of the major challenges encountered in conducting this present review was the large heterogeneity among the different included and excluded studies in terms of sample size, duration of studies, placebo and nonplacebo controls, methodology in assessing DH, lack of detail on randomization or allocation concealment, and so forth. Cummins [76] previously suggested that considerable variation existed in the design and conduct of DH studies before 1997 which could invariably introduce bias into a systematic review when different studies are compared. Studies conducted after 1997 generally follow the Holland et al. [69] guidelines when assessing various desensitizing agents [5] and one of the advantages of the studies using these guidelines is that this will enable the investigators to compare studies which have a similar methodology to evaluate a desensitizing agent.

The results from the small number of included calcium sodium phosphosilicate (CSPS) studies have limited any definitive conclusion on its efficacy in alleviating DH although it can be acknowledged from these studies that CSPS may have some effect on DH alleviation. A similar conclusion may also be applied to the efficacy of ACP containing toothpastes with only one included study in the present review.

None of the studies retrieved for inclusion in the present review directly compared the four selected four products using the same study design and methodology. Furthermore, there were no randomized controlled trials that compared at least two of the products in the present review. No conclusions can therefore be made from any direct comparison of CSPS, ACP, (nano)HAP, or ACP toothpastes and their efficacy on DH in the included studies.

No conclusions can be made from this review on the efficacy of any of the other selected two agents; (nano)HAP and CPP-ACP toothpastes due to the lack of sufficient evidence to support any efficacy in reducing DH.

The conclusions from the present review would therefore suggest that there is a paucity of well-conducted DH studies based on the Holland et al. [69] recommendations when conducting studies of this nature.

## Figures and Tables

**Table 1:** Characteristics of Novamin containing toothpastes excluded studies.

Number	Study	Reason
1	Bakry et al. [11]	*In vitro* investigation

2	Burwell et al. [12]	*In vitro* investigation

3	Burwell et al. [13]	*In vitro* investigation

4	Chiang et al. [14]	*In vitro* investigation

5	Earl et al. [15]	*In vitro* investigation

6	Farmakis et al. [16]	*In vitro* investigation

7	Gillam et al. [17]	*In vitro* investigation

8	Gjorgievska and Nicholson [18]	*In vitro* investigation

9	Golpayegani et al. [19]	*In vitro* investigation

10	Mitchell et al. [20]	*In vitro* investigation

11	Mneimne et al. [21]	*In vitro* investigation

12	Parkinson et al. [22]	Abstract only (at the time of collection of papers). However as the study lacked a negative control in the clinical trial design methodology it was excluded from the present review

13	Sauro et al. [23]	*In vitro* investigation

14	Wang et al. [24]	*In vitro* investigation

15	Wang et al. [25]	*In vitro* investigation

16	Greenspan [26]	Review

17	Du et al. [27]	No mention of randomisation and allocation concealment methods

18	Patsouri et al. [28]	Abstract only

19	Surve et al. [29]	Abstract only (at the time of collection of papers). However the study investigated the combination of an In-office application with or without a OTC desensitizing toothpaste following periodontal treatment and was therefore considered irrelevant for the present review

20	Yu et al. [30]	Publication in Chinese (Abstract in English)

21	Milleman et al. [31]	In-office agent (professionally applied polishing paste) and not as an OTC desensitizing toothpaste

22	Narongdej et al. [32]	Novamin used as in-office agent

23	Banerjee et al. [33]	Study duration ≤10 days

24	Tai et al. [37]	Investigated the antigingivitis effect of Novamin on the gingival tissues (desensitizing toothpaste *per se*) and therefore the study was considered irrelevant to this review

25	Acharya et al. [34]	Excluded as the publication date was after December 31st and lacked a negative control in the clinical trial design methodology

26	Ananthakrishna et al. [35]	Excluded as the publication date was after December 31st and lacked a negative control in the clinical trial design methodology

27	Neuhaus et al. [36]	Excluded as the publication date was after December 31st and evaluated an In-office agent (professionally applied polishing paste)

28	Salian et al. [38]	The negative control group contained fluoride whereas the test group (Novamin) did not

29	Sharma et al. [39]	The negative control group contained fluoride whereas the test group (Novamin) did not

30	West et al. [40]	The dentine specimens were placed in intraoral appliances worn by patients to establish an *in situ* effect of Novamin

31	Rajesh et al. [41]	One further study was excluded due to the lack of description on the composition of the control randomization

**Table 2:** Characteristics of ACP containing toothpastes excluded studies.

Number	Study	Reason
1	Thanatvarakorn et al. [42]	*In vitro* investigation examining the effects of ACP on dentine tubule occlusion
2	Tirapelli et al. [43]	*In vitro* investigation examining the effects of ACP on dentine tubule occlusion
3	Winston et al. [44]	*In vitro* investigation examining the effects of ACP on dentine tubule occlusion
4	Charig et al. [45]	*In vitro* investigation examining the effects of ACP on dentine tubule occlusion
5	Fiocchi et al. [46]	Excluded due to the investigators examining DH as a result of vital bleaching
6	Giniger et al. [47]	Excluded due to testing effect of agent on vital tooth bleaching
7	Geiger et al. [49]	Excluded due to the lack of a negative control (the control group was a KCl product)
8	Yates et al. [50]	Excluded since the ACP was applied as an in-office agent and not as an OTC toothpaste

**Table 3:** Characteristics of HAP containing toothpastes excluded studies.

Number	Study	Reason
1	Mukai et al. [51]	*In vitro* investigation and did not meet the inclusion criteria

2	Zhang et al. [52]	*In vitro* investigation and did not meet the inclusion criteria

3	Braun et al. [53]	*In vitro* investigation and did not meet the inclusion criteria

4	Lee et al. [54]	*In vitro* investigation and did not meet the inclusion criteria

5	Rimondini et al. [55]	*In vitro* investigation and did not meet the inclusion criteria

6	Yuan et al. [56]	*In vitro* investigation and did not meet the inclusion criteria

7	Tschoppe et al. [57]	*In vitro* investigation and did not meet the inclusion criteria

8	Akatsuka et al. [58]	*In vitro* investigation and did not meet the inclusion criteria

9	Shetty et al. [59]	Excluded an in-office agent rather than a toothpaste

10	Orsini et al. [60]	Excluded due to the lack of a negative control being used

11	Browning and Deschepper Maed [61]	Excluded due to the investigators examining the effect of HAP on DH following dental bleaching

**Table 4:** Characteristics of CPP-ACP containing toothpastes excluded studies.

Number	Study	Reason
1	Gandolfi et al. [62]	*In vitro* investigation investigating the effect of CPP-ACP on dentine tubule occlusion

2	Ranjitkar et al. [63]	*In vitro* investigation investigating the effect of CPP-ACP on dentine tubule occlusion

**Table 5:** Characteristics of Novamin containing toothpastes included studies.

Number	Studies	Methods	Participants	Interventions	Outcomes	Results
1	Pradeep et al. [64]	6-week triple-blinded RCT	149 completing out of 160	Group A: 5% potassium nitrate toothpaste -Group B: 5% calcium sodium phosphosilicate toothpaste -Group C: 3.85% amine fluoride toothpaste -Group D: placebo toothpaste (negative control; no information was provided as to the constituents of control)	Pre-op and post-op evaporative (controlled air stimulus) and thermal stimuli (10 mL of ice cold water) used	5% CSPS > 3.85% amine fluoride > KNO_3_ > placebo

2	Pradeep and Sharma [65]	6-week triple-blinded RCT	110 completing out of 120	Group A: 5% calcium sodium phosphosilicate (Novamin) toothpaste -Group B: 5% potassium nitrate toothpaste (positive control) -Group C: same formulation as toothpaste A; however, no active ingredient (negative control)	Pre-op and post-op evaporative (controlled air stimulus) and thermal stimuli (10 mL of ice cold water) used	5% CPS > KNO_3_ > placebo

3	Litkowski and Greenspan [66]	8-week double-blind randomized placebo controlled pilot study	66 No mention of dropouts	Group A: placebo control toothpaste (negative control; no information was provided as to the constituents of control) -Group B: 2.5% Novamin toothpaste -Group C: 7.5% Novamin toothpaste	Pre-op and post-op tactile (Yeaple probe calibrated at 40 g force) and an evaporative stimulus (one-second air blast)	7.5% CSPS > 2.5% CSPS > placebo

**Table 6:** Characteristics of ACP containing toothpastes included studies.

Number	Studies	Methods	Participants	Interventions	Outcomes	Results
	Ghassemi et al. [67]	8-week, parallel, double-blind RCT	203 of 208 participants completed trial	Group A: ACP and 0.24% NaF Group B: control toothpaste containing 0.24% NaF	Thermal: cold air blast (VAS and Schiff tests recorded)	ACP containing toothpaste > to control toothpaste

	Kaufman et al. [68]	8-week double-blind RCT	101 of 105 participants completed study	Group A: NaF conventional OTC control Group B: 1150 NaF and ACP Group C: 1150 ppm MFP and ACP	Electrical (sensitometer) Tactile (scratchometer) Evaporative (one-second air blast)	ACP and NaF > ACP > MFP > OTC control

**Table 7:** Comparison of the duration of the included studies.

Study	Duration
Pradeep et al. [64]	6 weeks

Pradeep and Sharma [65]	6 weeks

Litkowski and Greenspan [66]	8 weeks

Ghassemi et al. [67]	Phase I: 8 weeks
	Phase II: 8 weeks

Kaufman et al. [68]	8 weeks

**Table 8:** Comparison of dropouts from the included studies.

Study	Dropouts/withdrawal	Reason for withdrawal
Pradeep et al. [64]	11	No reason specified

Pradeep and Sharma [65]	10	No reason specified

Litkowski and Greenspan [66]	No mention of dropouts	No reason specified

Ghassemi et al. [67]	Phase I: 5	No reason specified
	Phase II: 13	

Kaufman et al. [68]	4	No reason specified
